# Solubilized Pancreatic Extracellular Matrix from Juvenile Pigs Protects Isolated Human Islets from Hypoxia-Induced Damage: A Viable Option for Clinical Islet Transplantation

**DOI:** 10.1155/2023/7452682

**Published:** 2023-07-11

**Authors:** Heide Brandhorst, Stasia Krishtul, Daniel Brandhorst, Limor Baruch, Marcelle Machluf, Paul R. V. Johnson

**Affiliations:** ^1^Nuffield Department of Surgical Sciences, University of Oxford, Oxford No. 3 9DU, UK; ^2^Laboratory for Cancer Drug Delivery and Cell Based Technologies, Faculty of Biotechnology and Food Engineering, Technion Israel Institute of Technology, Haifa, Israel; ^3^Oxford Biomedical Research Centre (OxBRC), Oxford No. 3 9DU, UK

## Abstract

The pancreatic extracellular matrix (ECM) is an enormously complex construct. Previous studies underline the challenges to identify the optimal combinations and ratios of individual ECM proteins for promoting survival and function of isolated and transplanted islets. This study aimed on assessing the efficiency of solubilized natural ECM extracted from juvenile pigs, an unlimited donor source. Isolated human islets were cultured under a hypoxic atmosphere (2% oxygen) in media supplemented with either solubilized porcine pancreatic ECM (ppECM) or a mixture of human ECM proteins composed of collagen-IV, laminin-521, and nidogen-1 (hEPM). Control islets were cultured under identical conditions without ECM-compounds. Reactive oxygen species production increased three-fold in controls but was reduced by hEPM or ppECM. Early apoptosis remained on preculture levels when islets were treated with hEPM or ppECM. Preculture viability was preserved when hEPM or ppECM was administered. Whilst controls failed to respond to glucose challenge, treatment with hEPM or ppECM preserved the physiological insulin response. In summary, overall survival was significantly highest in ppECM-treated islets. This study presents a new approach to protect human islets from hypoxia-induced damage by supplementing media with ppECM extracted from an unlimited donor source. The findings may also serve as starting point for a novel encapsulation technique to protect isolated human islets.

## 1. Introduction

The requirement to extract a subpopulation of heterogeneous cell clusters from an organ whilst preserving their native structure and organization is a unique challenge faced during pancreatic islet isolation compared with other types of tissue separation [1]. This requires a combination of enzymatic and mechanical techniques. During this process, the pancreatic matrix is digested, and the islets released from the dispersed acinar tissue before the two tissues are separated by density-gradient purification. A key aspect of islet extraction, therefore, is the enzymatic detachment or cleavage of the islet basement membrane from the surrounding pancreatic matrix [2]. As a consequence, the bidirectional communication between the integrins expressed on the islet cell surface and the basement membrane is lost [3–6]. This, in turn, interrupts the signaling of vital physiological functions from the proximal microenvironment to the islet cells suffering from increased rates of apoptosis and severe loss during islet culture [7, 8]. The absence of an extracellular matrix (ECM) [9, 10], in combination with hypoxic conditions [11] and a shortage of essential nutrients [12], is the key factor resulting in 73% of islet recipients currently requiring two or more islet grafts to achieve improved glycaemia and elimination of life-threatening hypoglycemia unawareness [13].

Numerous attempts have been made to identify an ECM substitute for islet culture. These have mainly involved in the use of individual ECM proteins [14–19]. However, a small number of studies used combinations of different ECM proteins and found a synergistic effect on islet function and viability when compared with individual ECM proteins [20, 21]. In contrast, our group observed no significant protection of isolated human islets when combining collagen type-IV (COL-4), Laminin-521 (L-521), and Nidogen-1 (NID-1) compared with using individual basement membrane proteins. In fact, we detected that a concentration-related detrimental impact on islet integrity was noted after combining ECM proteins [22]. Llacua et al. also had similar findings of harmful effects of overdosed ECM proteins in their study [23]. The importance of identifying the most suitable ratios and concentrations of ECM proteins was emphasised in another study where COL-4, L-521, and NID-1 had been combined [24]. As demonstrated by analyzing the pancreatic and islet matrisome, the pancreatic ECM is enormously complex [25, 26], and the data from all these studies underline the challenges of identifying the optimal combination of ECM proteins for promoting survival and function of isolated islets before and after transplantation. To achieve this, consideration of the correct composition, structure, and stoichiometric ratios of the pancreatic ECM components is required [27].

As a consequence of these requirements, the current gold standard of scaffolds for tissue-engineering that mimic the natural environment of cells is decellularized matrices [28]. Although human pancreatic tissue is deemed as ideal ECM source for protection of isolated human islets, the shortage of young healthy human organ donors is a viable argument to select other species as tissue donors, such as the pig, particularly when the production of hydrogel-related products is intended [29–31].

The aim of this study therefore was to compare the efficiency of a preassessed and effective combination of human COL-4, L-521, and NID-1 [22] to solubilized ECM manufactured from decellularized porcine pancreases, which represents the most relevant preclinical model and that is already in use for numerous FDA-approved clinical applications [28, 32].

## 2. Materials and Methods

**Figure 1:**
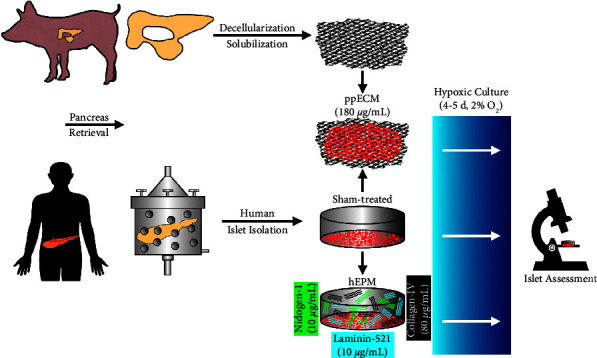
Experimental study design of comparing isolated human islets treated with either solubilized porcine pancreatic extracellular matrix (ppECM) or with a pretested mixture of human ECM proteins (hEPM) composed of collagen-IV, laminin-521, and nidogen-1.

An overview of the experimental study design is shown in Figure 1.

### 2.1. Manufacturing of Solubilized ECM from Porcine Pancreases

The manufacture of solubilized ECM from decellularized porcine pancreases (ppECM) was performed as previously described in detail [33]. Briefly, the pancreases were retrieved from 6 months old slaughterhouse pigs, trimmed, and dissected prior to alternating treatment with hyper- and hypotonic solution prepared as 1.1 and 0.7% (w/v) of sodium chloride dissolved in double-distilled water. Afterwards, the tissue was incubated at 37°C in 0.05% (w/v) of trypsin supplemented with 0.02% (w/v) of EDTA (Sigma-Aldrich, Rehovot, Israel) prior to a treatment with 1% (v/v) of Triton-X-100 (Sigma-Aldrich) and ammonium hydroxide in phosphate-buffered saline (PBS). Acellularity of the treated tissue was confirmed by hematoxylin and eosin staining of 10 *μ*m-tissue sections. After overnight lyophilization, 500 mg aliquots of ground extracellular matrix (ECM) were immersed in 20 mL of 0.1 M HCl supplemented with 100 mg of pepsin (Sigma-Aldrich) and stirred for 48 hours at room temperature. After solubilization had been completed, pepsin was inactivated by adjusting the pH to 7.4. Finally, the material was aliquoted (500 mg/mL) and stored at −20°C.

### 2.2. Islet Isolation and Culture

Eight human donor pancreases within a range 40 to 59 years were voluntarily donated with written consent according to the Declaration of Istanbul. Ethical approval for using isolated human islets for research was given by the NHS National Research Ethics Service (09/H0605/2). After a cold ischaemia time of 6.1 ± 1.6 hours (mean ± standard deviation [SD]), islets were isolated and purified as previously described [2]. After isolation, islets were cultured for 4-5 days in hypoxic atmosphere at 2% oxygen and 5% CO_2_. Aliquots of approximately 1000 (range 1300−700) islet equivalents (IEQ) were incubated per well in 12-well plates (Greiner Bio-One, Stonehouse, U.K.) and suspended in 800 *μ*L/well of CMRL 1066 supplemented with 20 mmol/L HEPES, 2 mmol/L L-glutamine, 200 units/mL penicillin, 200 *μ*g/mL streptomycin (all reagents from Life Technologies, Paisley, U.K.) and 2% fetal calf serum (PAA Laboratories, Pasching, Austria). In one of the treatment groups, ppECM, manufactured as described above, was added in a concentration of 180 *μ*g/mL in order to prevent the solidification of the ppECM into a hydrogel [34]. This experimental group was compared with islets cultured in the presence of a mixture of human ECM proteins (hEPM) assessed in a previous study [22] and composed of 80 *μ*g/mL COL-4 (Sigma-Aldrich, Dorset, U.K.), 10 *μ*g/mL L-521 (Biolamina, Uppsala, Sweden), and 10 *μ*g/mL NID-1 (R&D Systems, Abingdon, United Kingdom). Sham-treated islets, cultured under identical conditions in supplemented CMRL 1066 without the addition of any ECM-components, served as controls. All experimental groups were performed as free-floating culture.

### 2.3. Islet Characterization

After the culture in hypoxic atmosphere, islet-preconditioned supernatants were collected and assessed in duplicate for production of hypoxia- and inflammation-related chemokines. Release of tumor necrosis factor alpha (TNF-*α*), interleukin-1 beta (IL-1*β*), IL-6, IL-8, monocyte chemoattractant protein-1 (MCP-1), and vascular endothelial growth factor A (VEGF-A) was detected utilising enzyme immunoassays specific for human chemokines (Invitrogen/Thermo Fisher, Rochford, U.K.). After retrieval of the supernatants islets of a well were collected in a sample volume of 1 mL and assigned to a certain quality assessment parameter measured for the corresponding treatment group. All samples were measured in duplicate except the counting samples which were assessed in triplicate. Islets for *in vitro* function were collected from the counting samples.

Before and after islet culture, islet number was quantified and expressed as islet particle number (IN) and converted to islet equivalents (IEQ) as previously described in detail [35]. Islet yield, expressed as a percentage, was calculated by normalizing IEQ to preculture yield of IEQ. Islet morphological integrity was determined as a fragmentation index by calculating the ratio of IN over IEQ. Islet viability was assessed using 0.67 *μ*mol/L fluorescein diacetate (FDA, Sigma-Aldrich) and 4.0 *μ*mol/L propidium iodide (PI, Sigma-Aldrich) for staining of viable and dead cells, respectively [36]. Islet overall survival was used to calculate the recovery of living cells only. For this variable, normalized postculture islet yield was multiplied by the proportion of viable cells. Apoptosis in hypoxic islets was determined by simultaneous staining with Annexin-V FITC (Becton-Dickinson Biosciences, Oxford, U.K.) and PI used at a concentration of 450 ng/mL and 4.0 *μ*mol/L, respectively. Intraislet generation of reactive oxygen species (ROS) was determined in duplicate by measuring the intracellular conversion of dichlorofluorescein diacetate (DCFH-DA) into fluorescent dichlorodihydrofluorescein (DCFH) as previously described in detail [37].

*In vitro* function of 20 hand-picked islets of similar size (150–200 *μ*m) was assessed in duplicate during static glucose incubation as previously described in detail [38]. Islets were seeded on 8 *μ*m-pore size filter inserts, transferred into 24-well plates, and sequentially incubated for 45 min in 1 mL Krebs-Ringer buffer supplemented with 2.0 mmol/L glucose followed by 45 min at 20 mmol/L followed by a second period of 45 min at 2 mmol/L glucose. Afterwards, islets were recovered and sonicated in distilled water for disintegration prior to subsequent determination of DNA content measured by the Pico Green assay (Life Technologies). To minimize sample contamination with DNA released by dead and fragmenting cells, every sample was washed twice to carefully remove the supernatant with cell debris and released DNA. An aliquot of disintegrated islet cell suspension was mixed with acid ethanol at a ratio of 1 : 4 followed by overnight insulin extraction at 4°C. Prior to performance of the enzyme immunoassay samples were diluted and neutralized by Krebs-Ringer-buffer. Intracellular and secreted insulin was determined utilizing an enzyme immunoassay for human insulin (Mercodia, Uppsala, Sweden). Chemokine as well as insulin release was normalized to ng of DNA. Furthermore, insulin secretion was expressed as percentage of intracellular insulin content [39]. The glucose stimulation index (-----) was calculated by dividing the insulin release at 20 mmol/L glucose by the mean of the two basal periods. In order to evaluate the total secretory potency of an entire treatment group, the postculture recovery of IEQ was multiplied by the glucose stimulation index and expressed as proportion of sham-treated islets.

The fluorescence intensity (FI) of FDA, PI, Annexin-V, and DCFH was quantified in duplicate by means of a fluorometric plate reader as previously described [40]. As performed for the cytokine and insulin release and early apoptosis, ROS production was normalized to islet DNA content as well.

### 2.4. Statistical Analysis

Statistical analysis was performed using Prism 9.4.0 for MacIntosh (GraphPad, CA, USA). Data analysis was carried out by the nonparametric Friedman test followed by Dunn's test for multiple comparisons or by the Wilcoxon test. Differences were considered significant at *p* less than 0.05. *p* values more than 0.05 were deemed nonsignificant (NS). Results are expressed as mean ± standard deviation (SD) and are normalized to islet variables determined preculture if appropriate.

## 3. Results

**Figure 2:**
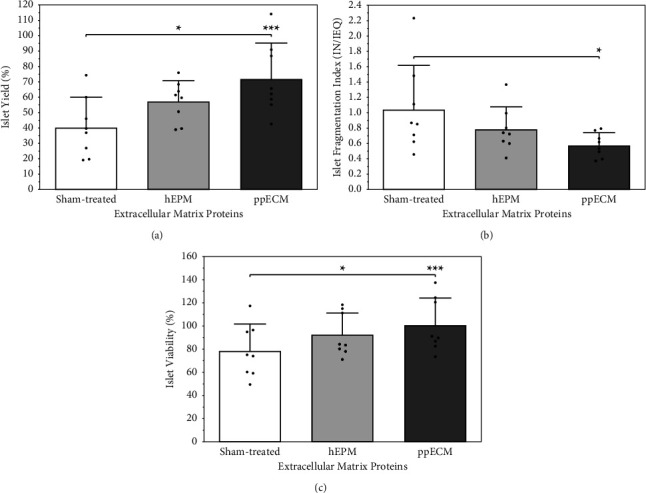
Effect of ECM proteins provided as mixture of three human ECM proteins (hEPM, light grey bars) or solubilized ECM produced from decellularized porcine pancreases (ppECM, dark grey bars) on (a) islet yield, (b) islet fragmentation, and (c) viability of human islets cultured for 4-5 days in hypoxic atmosphere (*n* = 8). (a, c) ^*∗*^*p* < 0.05 for sham-treated vs. hEPM, ^*∗∗∗*^*p* < 0.001 for sham-treated vs. ppECM. (b) ^*∗*^*p* < 0.05 for sham-treated vs. ppECM.

The major characteristics of human islet morphological integrity after culture in hypoxic atmosphere of 2% oxygen are shown in Figure 2(a)–2(c). Nearly 60% of the sham-treated islets were lost during 4-5 days of hypoxic culture (*p* < 0.001 vs. preculture) (Figure 2(a)). Nevertheless, when the islets were cultured in the presence of a mixture of human COL-4, L-521, and NID-1 (hEPM), the recovery was significantly increased (*p* < 0.05 vs. sham-treated;*p* < 0.01 vs. preculture). Islet recovery could be even further improved by adding ppECM to the culture medium (*p* < 0.001 vs. sham-treated; NS vs. preculture) (Figure 2(a)). Loss of islets during hypoxic culture was closely associated with an increase of the preculture fragmentation index of initially 0.44 ± 0.17 (*p* < 0.01 vs. sham-treated;*p* < 0.05 vs. hEPM) which was lowest in the ppECM group (NS vs. preculture) (Figure 2(b)). Another indicator of islet fragmentation is islet purity reflecting the accumulation of cell fragments and tissue debris during hypoxic culture. As demonstrated in Table 1, islet purity was found to be significantly higher when culture media were supplemented with hEPM or ppECM (NS vs. preculture; *p* < 0.01 vs. sham-treated).

Viability of sham-treated islets as reflected by FDA-PI staining was reduced during hypoxia by approximately 20% in average when normalized to the preculture islet viability of 66.0 ± 8.9% (*p* < 0.01 vs. sham-treated) (Figure 2(c)). In contrast, the presence of hEPM minimized the loss of viability in hypoxic islets (NS vs. preculture; *p* < 0.05 vs. sham-treated). Moreover, the addition of ppECM completely preserved the initial preculture viability during 4-5 days of hypoxia (NS vs. preculture; *p* < 0.001 vs. sham-treated). When simultaneously considering recovery and viability by calculating islet overall survival, it became obvious that this variable was significantly increased by adding hEPM (*p* < 0.05 vs. sham-treated) (Table 1). Furthermore, using ppECM nearly doubled the overall survival compared with sham-treatment (*p* < 0.001) and was also significantly more efficient in comparison with hEPM (*p* < 0.05).

**Figure 3:**
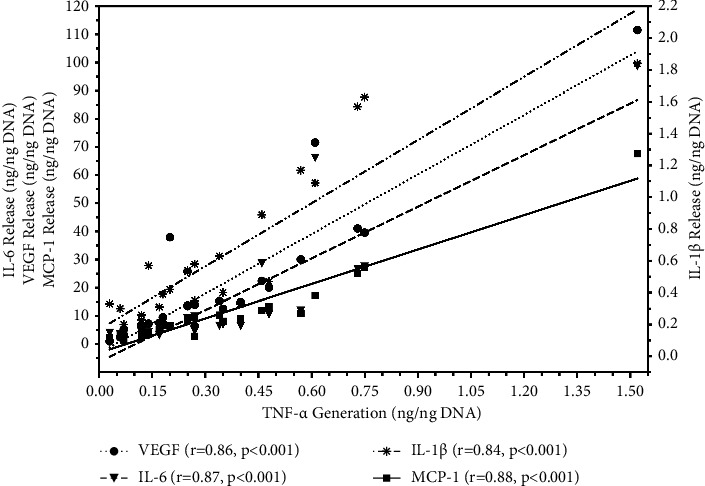
Correlation between islet TNF-*α* production (*n* = 24) in hypoxic atmosphere and generation of IL-1*β* (-••-••✳••-••-, right *y*-axis), IL-6 (– –▼– –, left *y*-axis), MCP-1 (––_■_––, left *y*-axis), and VEGF-A (•••●•••, left *y*-axis). The correlation coefficient (r) was calculated using Spearman's rank correlation test.

As expected, the lack of oxygen during culture triggered a proinflammatory state in hypoxic islet cells as reflected by the production of 6 different chemokines. As shown in Table 2, sham-treated islets released the significantly highest levels of chemokines (*p* < 0.01 vs. hEPM; *p* < 0.05–*p* < 0.01 vs.ppECM). Despite the differences between the amounts of individual chemokines that were produced, the accumulated release of the different chemokines followed a very similar pattern. In general, the chemokine production in the treatment groups was at least 50% lower compared with sham-treated islets. The close interrelationship between the individual chemokines is also reflected by the high correlations that were found between TNF-*α*, the central and dominant component of the chemokine network, and IL-1*β*, IL-6, MCP-1, and VEGF-A as shown in Figure 3. The calculated correlation coefficient was always *r* ≥ 0.80. This applies also to IL-8 which is not included in Figure 3 (*r* = 0.80; *p* < 0.001).

A tight correlation was also detected between TNF-*α* and ROS (*r* = 0.90, *p* < 0.001) which serve as signaling molecules for TNF-*α* activity [41]. As demonstrated in Table 1, the intraislet generation of ROS in sham-treated islets increased almost threefold during hypoxic culture when compared with the preculture level of ROS production (21.4 ± 15.8 FI/ng DNA, *p* < 0.001 vs. sham-treated). The ROS production in hEPM-treated islets reached approximately only half of that measured in sham-treated islets (NS vs. preculture; *p* < 0.01 vs. sham-treated). However, when hypoxic islets were cultured in the presence of ppECM, the level of ROS generation could be stabilized on the preculture level (NS vs. preculture; *p* < 0.01 vs. sham-treated).

Although the differences between the experimental groups in terms of islet viability were significant, the discrepancy between sham-treated islets and the treatment groups was considerably more distinct with respect to apoptosis (Table 1). Remarkably, the extent of apoptosis in islets cultured in the presence of ppECM was significantly lower when compared with the preculture level of apoptosis (19.5 ± 13.0 FI/ng DNA, NS vs. hEPM; *p* < 0.05 vs. sham-treated, vs. ppECM).

**Figure 4:**
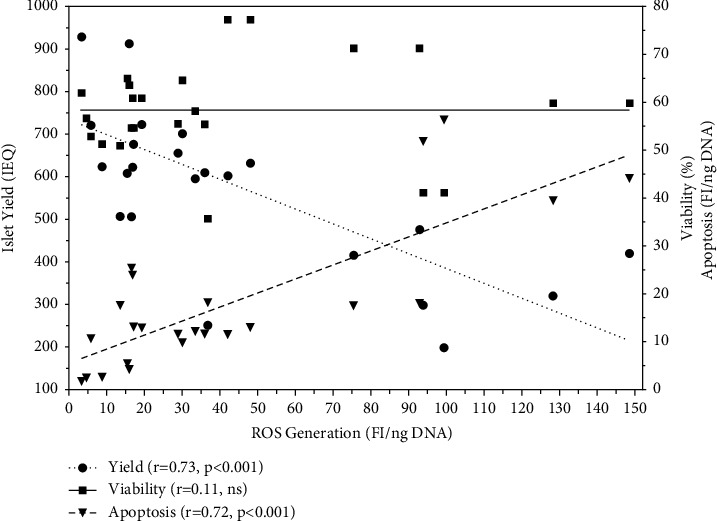
Effect of islet ROS production (*n* = 24) in hypoxic atmosphere on postculture islet yield (**•••**_**●**_**•••**, left *y*-axis), viability (––_■_––, right *y*-axis), and apoptosis (**– –**_**▼**_**– –**, right *y*-axis). The correlation coefficient (r) was calculated using Spearman's rank correlation test.

In order to estimate the influence of ROS on different variables of islet cell death, matrix correlation analysis was performed. As shown in Figure 4, the analysis revealed that islet ROS production had a strong inverse effect on postculture islet yield (*r* = −0.73, *p* < 0.001) but did surprisingly not show any correlation with islet viability (*r* = 0.11, NS). A significant correlation was found between ROS and apoptosis (*r* = 0.72, *p* < 0.001). A similar observation was made for the production of TNF-*α* that correlated with apoptosis (*r* = 0.60, *p* < 0.01) and postculture islet yield (*r* = −0.59, *p* < 0.001) but no correlation was seen with islet viability (*r* = −0.01, NS). Other proinflammatory cytokines, such as IL-1*β*, IL-6, IL-8, and MCP-1, followed the same pattern as TNF-*α* regarding their correlation with islet yield, apoptosis, and islet viability (data not shown). Even VEGF, a representative of islet-protective chemokines, showed a similar correlation with islet yield (*r* = −0.59, *p* < 0.01), apoptosis (*r* = 0.62, *p* < 0.01), and islet viability (*r* = 0.15, NS).

**Figure 5:**
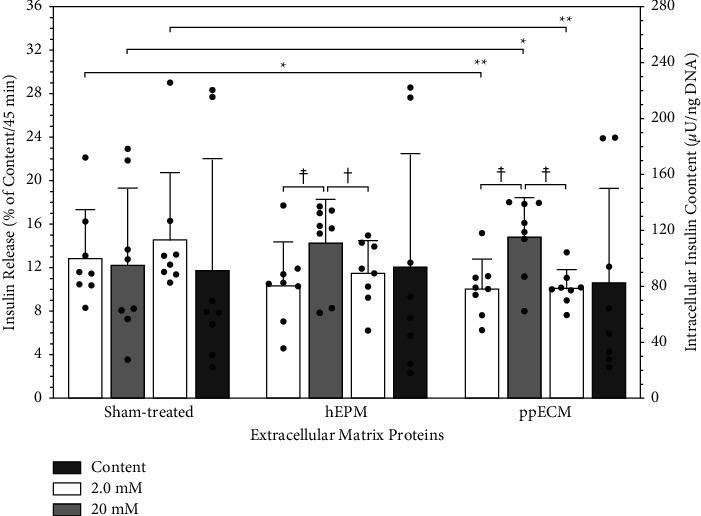
Glucose-stimulated insulin release after 4-5 days of human islet culture in hypoxic atmosphere (*n* = 8). Basal (white bars) and stimulated (grey bars) insulin release of 20 human islets of equal size is normalized to ng of islet DNA and expressed as percentage of intra-islet insulin content also normalized to ng of islet DNA. ^*∗*^*p* < 0.05 for sham-treated vs. hEPM. ^*∗*^*p* < 0.05, ^*∗∗*^*p* < 0.01 for sham-treated vs. ppECM as indicated. ^**†**^*p* < 0.05, ^**☨**^*p* < 0.01 for 2.0 vs. 20 mmol/L of glucose.

As seen in several previous studies, the lack of oxygen during 4-5 days of culture had a strong inhibitory effect on glucose-stimulated insulin release when islets were cultured without the presence of ECM-components. As demonstrated in Figure 5, sham-treated islets had a significantly higher basal insulin release than islets exposed to hEPM (*p* < 0.05) or ppECM (*p* < 0.01). In addition, sham-treated islets failed to up-regulate insulin release after glucose challenge and also failed to down-regulate insulin release after switching from high to low glucose concentration (*p* < 0.01 vs. ppECM). As a consequence, the glucose stimulation index dropped below the clinically relevant threshold of 1.0 [42] in contrast to islets treated with hEPM (*p* < 0.05 vs. sham-treated) or ppECM (*p* < 0.001 vs. sham-treated) (Table 1). In contrast to insulin release, intracellular insulin content was not significantly altered by treatment with hEPM or ppECM (Figure 5). When estimating the secretory potency of the experimental groups, a parameter that considers not only the stimulatory capacity but also the postculture recovery of islets, it became clear that islet treatment with hEPM (*p* < 0.05) or ppECM (*p* < 0.001) roughly triplicated the total secretory potency in comparison with sham-treated islets (Table 1).

## 4. Discussion

To the best of our knowledge, this is the first study that compares the protective potency of solubilized ECM from decellularized porcine pancreas (ppECM) on isolated human islet to a mixture of human COL-4, L-521, and NID-1 (hEPM). Under certain biochemical conditions, these ECM proteins assemble to form suprastructures [43, 44] that represent the most relevant constructs in the native human islet basement membrane [45]. In comparison with sham-treated islets, the mixture of human ECM proteins (hEPM) significantly protected the functional and structural integrity of human islets exposed to hypoxia with respect to yield, viability, and insulin secretory capacity. Nevertheless, islet protection was further increased by culturing hypoxic islets in the presence of ppECM. The extent of protection clearly correlates with the complexity of the materials. The mixture of COL-4, L-521, and NID-1 may potentially serve as a substitute for the enzyme-cleaved islet basement membrane, provided that the ideal ratio between the individual components can be identified [21, 22, 46]. In contrast, the physiological function of the ECM can be attributed to its hierarchical and orchestrated organization rather than to its individual components [45]. It is, therefore, very unlikely that the abundance of functions can be fully replaced by individual ECM components or limited constructs [34]. The same applies to synthetic compounds, despite the fact that their characteristics and properties can be tightly controlled [47–49]. In contrast, decellularized ECM provides a nearly endless variety of signals that are required to maintain tissue and cell integrity, growth as well as differentiation thus representing the current gold standard of scaffolds for tissue engineering that mimic the natural environment of cells [27].

Though the ideal source for such an ECM supplement for human islet protection postisolation would be a human source, the low availability of young healthy human pancreas donors is a substantial argument to consider other species as tissue donors. Porcine ECM from different organs has been shown to be a safe choice for clinical application, conserving its original bioactivity through the different species while evading an immune reaction. As such, the application of porcine ECM in diverse biomedical devices has been extensively investigated, including several FDA-approved clinical applications [28, 32].

The composition of decellularized ppECM was addressed in our previous work [50], revealing that the ppECM is a complex mixture, comprised of 84% collagen. Particularly, type I and type III collagen are the most abundant, accounting for 98% of the ppECM proteins, while other types of collagen, including II, V, VI, and IV are present in lower quantities. Proteins such as fibronectin, laminin, and others are also present in very low percentages but, apparently, highly contribute to the molecular structure and the biological activity of the material, thus equipping it with pancreas-specific attributes. In accordance, a quantitative histological comparison with other species revealed a lower periislet expression of collagen-IV, Laminin, and Fibronectin in the pig than observed in human pancreases [51]. Nevertheless, compared with the hEPM treatment in the present work (human collagen-IV, Laminin-521, and Nidogen-1), only collagen type IV (COL-4) is present in both treatments in different concentrations.

The interspecies biocompatibility of the solubilized ppECM, used in our study, can be demonstrated by the protection of human islet morphological integrity in a hypoxic environment and by a substantially decreased production of chemokines and ROS when compared with sham-treated islets. The reduction of proinflammatory mechanisms has also implications for the functional integrity of isolated islets particularly when islets had been exposed to a hypoxic environment. This not only concerns islet *in vitro* function but may also be of importance for islet posttransplant function. As discussed above, the enormous variety of prosurvival signals provided by the whole ECM may be one reason that different stages of cell death could be prevented or ameliorated by ppECM. Apart from its antiinflammatory effect, we found that ppECM minimized apoptosis to a level that was significantly lower compared with preculture values. Interestingly, ROS, as well as TNF-*α*, significantly correlated with apoptosis and islet yield but did not show any correlation with islet viability. This is consistent with the findings of previous studies showing that ROS are serving as signaling molecules for TNF-*α* [41, 52]. It has been demonstrated in isolated human islets that cytokines can induce apoptosis but are not the primary triggers of cell necrosis [53, 54]. Remarkably, VEGF, generally categorized as a survival factor for human islets in a detrimental environment [55], followed the same pattern as TNF-*α* by correlating positively with apoptosis and inversely with islet yield. This can be explained by the decisive role that is played by TNF-*α* in the cytokine network controlling the release of IL-8 and VEGF amongst other chemokines [56].

However, when considering islet overall survival the utilization of ppECM was significantly more effective than the triple mixture of human ECM proteins indicating that the manufacturing process of ppECM resulted in a product that provides a human islet-compatible environment. The effective application of ppECM may be partially attributed to its use as a media supplement, rather than coating culture plastic containers with this material. It was previously shown, that the adsorption of ECM structures to synthetic surfaces can significantly alter conformation and bioactivity of adsorbed ECM [57].

Nevertheless, the final proof of efficiency by presenting data from transplant experiments was not provided which clearly is a limitation of our study. In vivo studies would also reveal how long the protective effect of human islet pretreatment with ECM proteins may last after implantation. So far, we can only speculate whether the positive effect on human islets' functional and morphological integrity may improve survival at least during the early phase of islet engraftment.

## 5. Conclusion

This study shows that solubilized ECM produced from decellularized porcine pancreases is highly efficient for protecting isolated human islets from hypoxically induced cell damage. The interspecies biocompatibility of ppECM is demonstrated by its inhibitory effect on proinflammatory and proapoptotic mechanisms in hypoxic human islets, which exceeds the protective potency of a mixture of three human human islet basement proteins suggesting ppECM as viable alternative to limited combinations of human ECM proteins. Nevertheless, transplant experiments have to be undertaken in the future to provide the final proof of ppECM efficiency.

## Figures and Tables

**Table 1:** Islet characterization after 4-5 days of islet culture in hypoxic atmosphere (*n* = 8).

Treatments	Purity (%)	Overall survival (%)	ROS (FI/ng DNA)	Apoptosis (FI/ng DNA)	Stimulation index (20/2 mmol/L)	Total secretory potency (%)
Sham-treated	72.8 ± 15.1	33.6 ± 19.5	73.2 ± 51.1	29.5 ± 20.7	0.86 ± 0.33	100 ± 0
hEPM	89.0 ± 13.8^‡^	53.3 ± 16.1^†,§^	33.1 ± 32.6^‡^	13.9 ± 7.4^†§^	1.32 ± 0.21^†^	302 ± 201^†^
ppECM	86.7 ± 17.0^‡^	72.3 ± 27.4^#^	22.7 ± 16.1^‡^	11.0 ± 6.6^#^	1.47 ± 0.33^#^	385 ± 218^#^

^†^ *p* < 0.05, ^‡^*p* < 0.01, ^#^*p* < 0.001 vs. sham-treated;^§^*p* < 0.05 vs. ppECM; data are shown as mean ± SD.

**Table 2:** Islet chemokine production (ng/ng DNA) during 4-5 days of islet culture in hypoxic atmosphere (*n* = 8).

Treatments	TNF-*α*	IL-1*β*	IL-6	IL-8	MCP-1	VEGF-A
Sham-treated	0.61 ± 0.43	1.33 ± 1.31	30.6 ± 34.4	96.6 ± 79.1	20.9 ± 20.9	39.1 ± 36.5
hEPM	0.20 ± 0.14^‡^	0.38 ± 0.23^‡^	7.6 ± 8.7^‡^	43.6 ± 39.6^‡^	5.7 ± 3.3^‡^	12.7 ± 12.0^‡^
ppECM	0.25 ± 0.17^‡^	0.50 ± 0.30^‡^	7.2 ± 3.1^†^	46.6 ± 37.9^†^	6.9 ± 3.5^‡^	12.5 ± 8.7^‡^

^†^ *p*  <  0.05, ^‡^*p*  <  0.01 vs. sham-treated; data are shown as mean ± SD.

## Data Availability

The data used to support the study are available from the corresponding author upon reasonable request.
